# High-Performance Riveted Complementary-Structure Rotating Triboelectric Nanogenerator for Energy Harvesting from Slow-Speed Water Flows

**DOI:** 10.3390/ma19173569

**Published:** 2026-08-22

**Authors:** Bao Yang, Chang Peng, Zihao Wang, Fuwang Zhao, Licheng Zhou, Zhenyu Jiang, Yiping Liu, Liqun Tang, Zejia Liu, Jinli Piao

**Affiliations:** 1 Department of Engineering Mechanics, School of Civil Engineering and Transportation, South China University of Technology, Guangzhou 510640, China; byang20210415@scut.edu.cn (B.Y.); 202310181479@mail.scut.edu.cn (C.P.); pengchangzzzi@gmail.com (Z.W.); ctlczhou@scut.edu.cn (L.Z.); zhenyujiang@scut.edu.cn (Z.J.); tcypliu@scut.edu.cn (Y.L.); lqtang@scut.edu.cn (L.T.); 2 School of Marine Science and Technology, Northwestern Polytechnical University, Xi’an 710072, China; zhaofuwang@nwpu.edu.cn; 3 School of Fashion and Textiles, The Hong Kong Polytechnic University, Kowloon, Hong Kong SAR, China

**Keywords:** energy harvesting, riveted complementary structural design, rotating triboelectric nanogenerator, slow-speed water-flow-driven

## Abstract

Triboelectric nanogenerators (TENGs) are promising for harvesting low-frequency mechanical energy, but rotating TENGs (R-TENGs) driven by low-speed water flow remain constrained by limited driving torque, sliding-contact losses, and rotating-system stability. Here, a three-dimensional (3D) riveted complementary-structure rotating triboelectric nanogenerator (RCSR-TENG) is proposed for low-speed water-flow energy harvesting. A semi-analytical formulation incorporating a force-dependent real-contact fraction is developed to describe the coupled relationships among output voltage, transferred charge, rotation angle, and contact force. Because the contact parameters were not independently calibrated, the formulation is used for sensitivity and trend analysis rather than as a quantitatively validated predictive model. For the single prototype tested for each configuration, at 1000 rpm under the fixed effective measurement load of 9 MΩ, the RCSR-TENG produced a peak output power of 544 μW, compared with 304 μW for the flat R-TENG, representing an increase of approximately 79%. The same RCSR-TENG prototype maintained a stable voltage amplitude of over 150,000 rotation cycles. When coupled to a fully passive flapping-foil collector in a 0.55 m s^−1^ water flow, the system generated periodic electrical output with a peak area-normalized power exceeding 5000 μW m^−2^. These results demonstrate the structural-performance advantage of the riveted complementary design and its proof-of-concept applicability to low-speed water-flow energy harvesting.

## 1. Introduction

Triboelectric nanogenerators (TENGs) convert mechanical energy into electrical energy through the coupled effects of contact electrification and electrostatic induction [1,2,3]. Since their first demonstration in 2012 [4], TENGs have attracted considerable attention because of their high output voltage, lightweight construction, cost-effectiveness, environmental compatibility, and ability to harvest low-frequency mechanical energy, typically in the range of 0.5–5 Hz [5,6,7,8]. According to the relative motion between the triboelectric interfaces, TENGs can be broadly classified into vertical contact-separation (CS) and lateral-sliding (LS) modes [9]. CS-mode TENGs operate through periodic normal contact and separation and consequently generate discrete high-voltage pulses. By contrast, rotating triboelectric nanogenerators (R-TENGs) generally operate in the LS mode, in which continuous rotation produces a periodic variation in the effective contact area [10,11,12,13]. This operating configuration enables sustained alternating electrical output and is readily coupled with continuous rotary motion, making R-TENGs promising for harvesting mechanical energy from hydrodynamic sources such as flowing water and ocean waves [14,15,16].

Low-speed water flow is widespread in natural environments but provides only a limited driving force, making high-speed rotation difficult to achieve. Considerable effort has therefore been devoted to improving the electrical output of R-TENGs under restricted mechanical input. Introducing supplementary charge between conventional R-TENG electrodes increased the output voltage by approximately threefold and increased both the current and transferred charge by approximately fivefold [17]. Another strategy is to combine compliant contact structures with appropriate triboelectric materials to increase the real contact area while reducing frictional losses. For example, a double-layer elastic R-TENG developed for harvesting wind energy from high-speed trains required only approximately half the driving torque of a conventional rotating-sliding TENG at the same electrical output, corresponding to the efficiency improvement defined in the cited study [18]. Although these approaches improve electrical performance by regulating interfacial charge or contact behavior, the available contact area within the limited device volume remains insufficiently utilized.

The electrical output of an R-TENG is strongly governed by the effective surface-charge density of its triboelectric layers. This effective charge density depends on the real contact conditions, including the real contact area, contact stress, relative sliding velocity, material properties, and environmental conditions. Continuous sliding may also cause surface scratching, mechanical wear, and vibration, leading to material degradation and reduced durability [19,20]. Replacing sliding contact with a rolling-contact structure can reduce interfacial wear and improve durability [21]. Alternatively, long-lived triboelectric surface charges can support electrostatic induction under non-contact operation. A disk-structured TENG operating in this mode maintained charges on its fluorinated ethylene propylene (FEP) film for several days and required only a small driving force, making it suitable for harvesting weak and irregular mechanical energy [22]. However, gradual charge dissipation without continuous charge replenishment can reduce its long-term electrical output. These considerations indicate that the contact interface must simultaneously provide sufficient charge generation, low mechanical resistance, and adequate durability.

Three-dimensional hierarchical surface structures offer a direct means of increasing the real contact area within a fixed device footprint. Structures containing both macroscopic features and microscopic textures can provide a large specific surface area and thereby promote interfacial contact. Riveted complementary structures are particularly attractive because they combine structural integrity, accurate assembly, effective load distribution, resistance to vibration and impact, and long-term mechanical stability. Although surface-morphology modification has been extensively investigated in CS-mode TENGs, the rational design of three-dimensional complementary surfaces for R-TENGs remains limited [23]. A suitable three-dimensional structure must not only increase the available contact area but also maintain smooth rotation and avoid excessive friction and wear.

The development of such structures also requires a theoretical description that connects three-dimensional contact behavior with electrical output. Existing R-TENG models primarily describe the relationships among output voltage, transferred charge, and rotation angle [24,25,26]. When a three-dimensional contact structure deforms under load, however, the real contact area changes and thereby alters the effective surface-charge density and electrical response. The present semi-analytical formulation therefore incorporates a force-dependent real-contact fraction into the conventional voltage–transferred-charge–rotation-angle relationship [27,28]. Because the contact parameters were not independently identified, the formulation is used for sensitivity and trend analysis rather than as a quantitatively validated predictive model. In addition, numerical simulation is required to examine edge-field effects when the triboelectric regions approach complete separation.

Beyond harvesting the macroscopic kinetic energy of wind, water flow, and ocean waves, the molecular-scale regulation of water–material interactions provides another route for improving microscale energy systems. In conjugated polymers, ion insertion and extraction are generally accompanied by hydration water, which affects ion transport, volumetric deformation, mechanical actuation, and hydrolytic degradation. The regulation of anion hydration has recently been used to suppress excessive water ingress into polyaniline, improve the stability of a submillimeter-scale micro battery, and reduce the energy consumption of a polypyrrole actuator, thereby enabling monolithic integration of energy storage and actuation [29]. Systematic investigation of π-conjugated polyelectrolytes has further shown that reversible hydration occurs predominantly at the interface between ionic clusters and the surrounding hydrophobic polymer matrix [30]. In ion-conducting polymer membranes, positioning hydrophobic pendant groups near charged sites can restrain hydration-induced pore swelling, regulate hydrated micropore dimensions, and improve ion selectivity while maintaining efficient ion transport in aqueous electrochemical devices [31]. Operando characterization of an n-type ladder organic mixed ionic-electronic conductor has additionally revealed that strong interactions between protic cations and the polymer backbone can disrupt ion hydration and expel water from the polymer at high doping levels, resulting in an unusual deswelling response rather than conventional hydration-induced expansion [32].

These developments establish water content, ion solvation, and hydration-induced structural evolution as active materials-design variables rather than passive environmental effects. Hydration control should nevertheless be distinguished from hydrodynamic energy harvesting. It does not provide an additional source of mechanical energy but instead regulates ion transport, energy storage, mechanical actuation, and interfacial stability. This distinction is particularly important for water-exposed micro- and nanogenerators, in which uncontrolled hydration may influence charge transport, mechanical deformation, material degradation, and long-term reliability. A practical water-flow energy system should therefore combine efficient hydrodynamic energy capture with controlled water–material interactions in its downstream storage, actuation, and sensing components.

A complementary development is the integration of micro- and nanogenerators with three-dimensional electronic devices and sensors. Compared with conventional planar layouts, three-dimensional architectures can increase the functional area within a limited footprint, shorten electrical connections between energy and sensing components, and form conformal interfaces with complex biological or environmental systems. Litho-graphically patterned thin-film stacks have been transformed into self-assembled coaxial Swiss-roll microfluidic devices containing integrated electrodes and a tunable polyimide proton-exchange membrane, demonstrating a compact three-dimensional architecture for bioelectronic power generation [33]. Three-dimensional five-directional braided triboelectric structures have also been developed to combine biomechanical energy harvesting with pressure and vibration sensing in resilient and shape-adaptable electronic textiles [34]. A triboelectric–electromagnetic hybrid nanogenerator has further been integrated with power-management electronics, intermediate energy storage, environmental sensors, signal-processing circuits, and wireless communication [35]. Three-dimensional printing has also been used to fabricate a soft robotic gripper containing integrated triboelectric deformation and tactile sensors, illustrating how three-dimensional manufacturing can combine mechanical actuation, self-powered sensing, and information processing within a common structural platform [36].

These advances demonstrate that three-dimensional geometric assembly must be accompanied by electrical and functional integration. A practical nanogenerator-powered sensor should therefore be considered as a coordinated system comprising energy harvesting, rectification, impedance matching, buffered storage, voltage regulation, signal acquisition, and data transmission. For water-flow energy harvesters, further requirements include waterproof electrical interconnections, corrosion and biofouling resistance, low-leakage power-management circuits, and mechanical isolation between the generator and sensitive electronic components. Three-dimensional integration can reduce the overall device footprint and connection length, but its benefits must be balanced against fabrication yield, structural variability, encapsulation complexity, and the possible influence of integrated electronics on the dynamics of the energy-harvesting structure.

To address these challenges, this study develops an R-TENG with three-dimensional riveted complementary surface structures, termed the RCSR-TENG. A semi-analytical formulation incorporating a force-dependent real-contact fraction is derived to support interpretation of the device’s behavior. The primary contribution is the structural design and its experimental evaluation, whereas the formulation is used to examine theoretical trends and is not presented as an independently calibrated predictive model. The influence of the complementary structures is investigated through theoretical analysis, comparative numerical simulation, geometric optimization, and experimental characterization. Finally, the RCSR-TENG is coupled with a fully passive flapping foil to demonstrate proof-of-concept energy harvesting from low-speed water flow.

## 2. Semi-Analytical Formulation Incorporating a Force-Dependent Real-Contact Fraction

**Figure 1:**
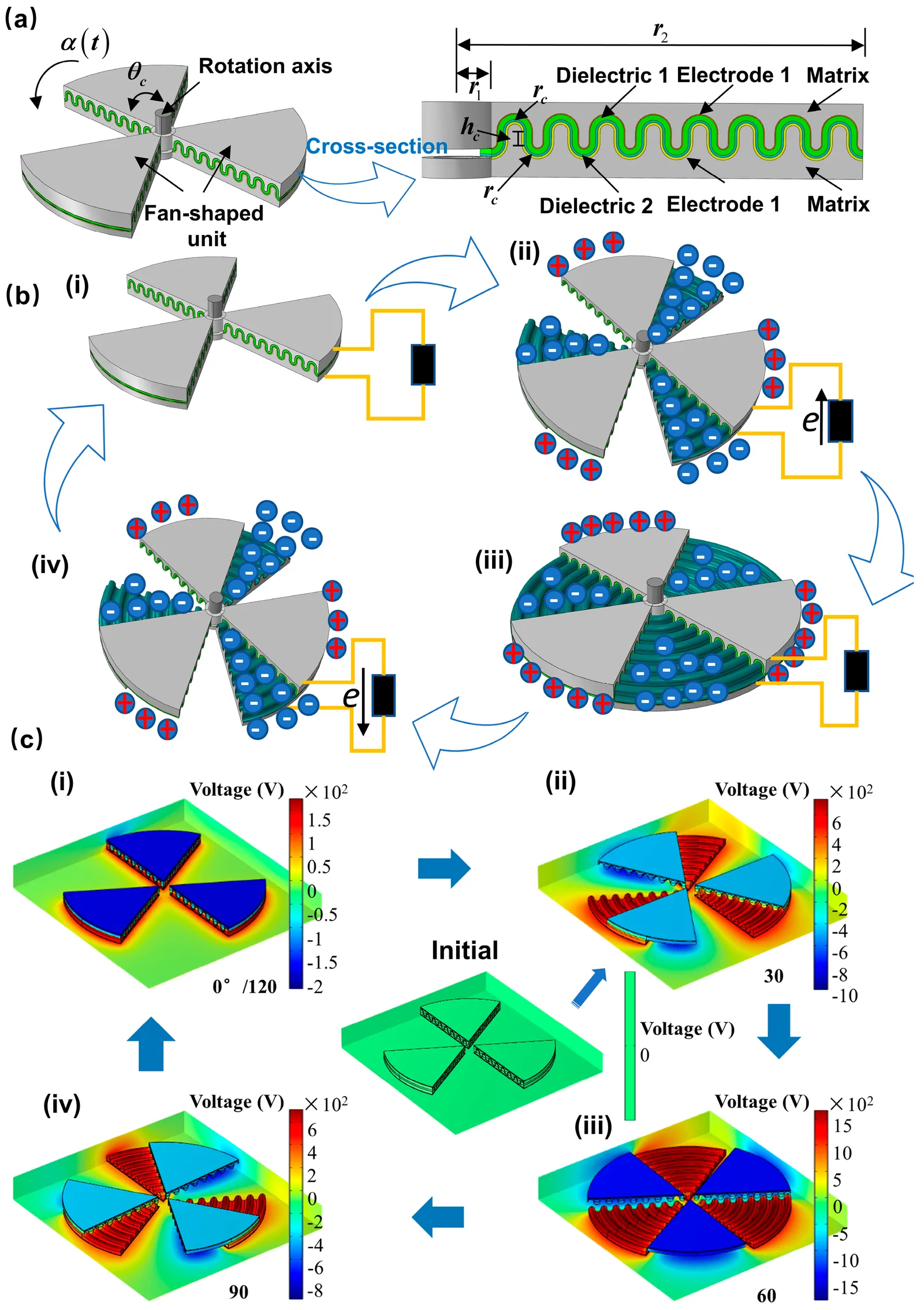
Structure and working principle of the RCSR-TENG. (**a**) Device structure. (**b**) Operating states: (i) full contact, (ii) partial separation, (iii) full separation, and (iv) partial re-contact. (**c**) Corresponding electric-potential distributions under open-circuit conditions. (i) full contact, (ii) partial separation, (iii) full separation, and (iv) partial re-contactThe RCSR-TENG operates through contact electrification and electrostatic induction. Repeated contact and relative sliding generate equal and opposite triboelectric charges on the two facing surfaces; after sufficient cycling, the surface-charge density approaches a steady value. In state (i), the upper and lower triboelectric layers are fully overlapped, and no instantaneous current flows through the external circuit. As the rotor moves toward state (ii), the decreasing overlap changes the induced electrode potential and drives electrons through the external load. In state (iii), the two patterned regions reach their minimum overlap, and the transferred charge reaches its maximum magnitude. As the rotor proceeds toward state (iv), the overlap increases again, reversing the direction of electron flow through the external load. Continuous rotation therefore produces a periodic alternating electrical output. Figure 1c shows representative electric-potential distributions obtained from COMSOL (6.0.2.339) electrostatic simulations at selected rotation angles. Starting from the fully over-lapped configuration, the open-circuit potential difference increases as the overlap decreases, reaches a maximum near the minimum-overlap state, and then returns toward zero as the next electrode sector becomes fully overlapped.

Figure 1a shows a schematic of the RCSR-TENG. The cylindrical device consists of two plates divided into fan-shaped units at equal angular intervals. One plate is fixed as the stator, whereas the other rotates as the rotor. Each unit comprises complementary three-dimensional features, triboelectric layers, electrode layers, substrate layers, and a central rotation axis. The complementary features serve two distinct functions: they increase the available interfacial contact area—which can promote charge generation—and they act as circumferential guidance tracks that stabilize rotor motion. The general configuration can operate in either dielectric-to-dielectric or conductor-to-dielectric contact mode; in the latter case, the thickness of dielectric layer 1 is set to zero in the model.

The contact profile is constructed by arranging repeated semicircular arcs and straight-line segments around the rotation axis. The derivation of the nominal contact areas of the RCSR-TENG and the flat-plate R-TENG is provided in Section S1 of the Supporting Information. The rotation-dependent nominal contact area is expressed as follows:

(1) $$A\left(t\right)=A_{m}\left[1-\frac{1}{\pi }\arccos\left(\cos\left(N\cdot \alpha \left(t\right)\right)\right)\right]$$

where $A_{m}=\frac{\pi }{2}\left[\left(r_{1}+\frac{\pi }{2}r_{2}-\frac{\pi }{2}r_{1}+n_{l}h_{l}\right)r_{2}-r_{1}^{2}\right]$—meaning the maximum nominal contact area. $r_{1}$ and $r_{2}$ are the inner and outer radius of the fan-shaped unit, respectively. $\alpha \left(t\right)$ is the rotation angle. $r_{c}$ and $h_{l}$ represent the radius of semi-circulars and the length of line segments, respectively. $n_{l}$ represents the number of line segments. As shown in Figure S1, the contact area can be up to several times higher than that of contact area of flat plates through adjusting radius of semi-circulars and the length of line segments. Larger values of $h_{l}$ lead to larger contact area. Meanwhile, with decreasing $r_{c}$, contact area also significantly increased.

**Figure 2:**
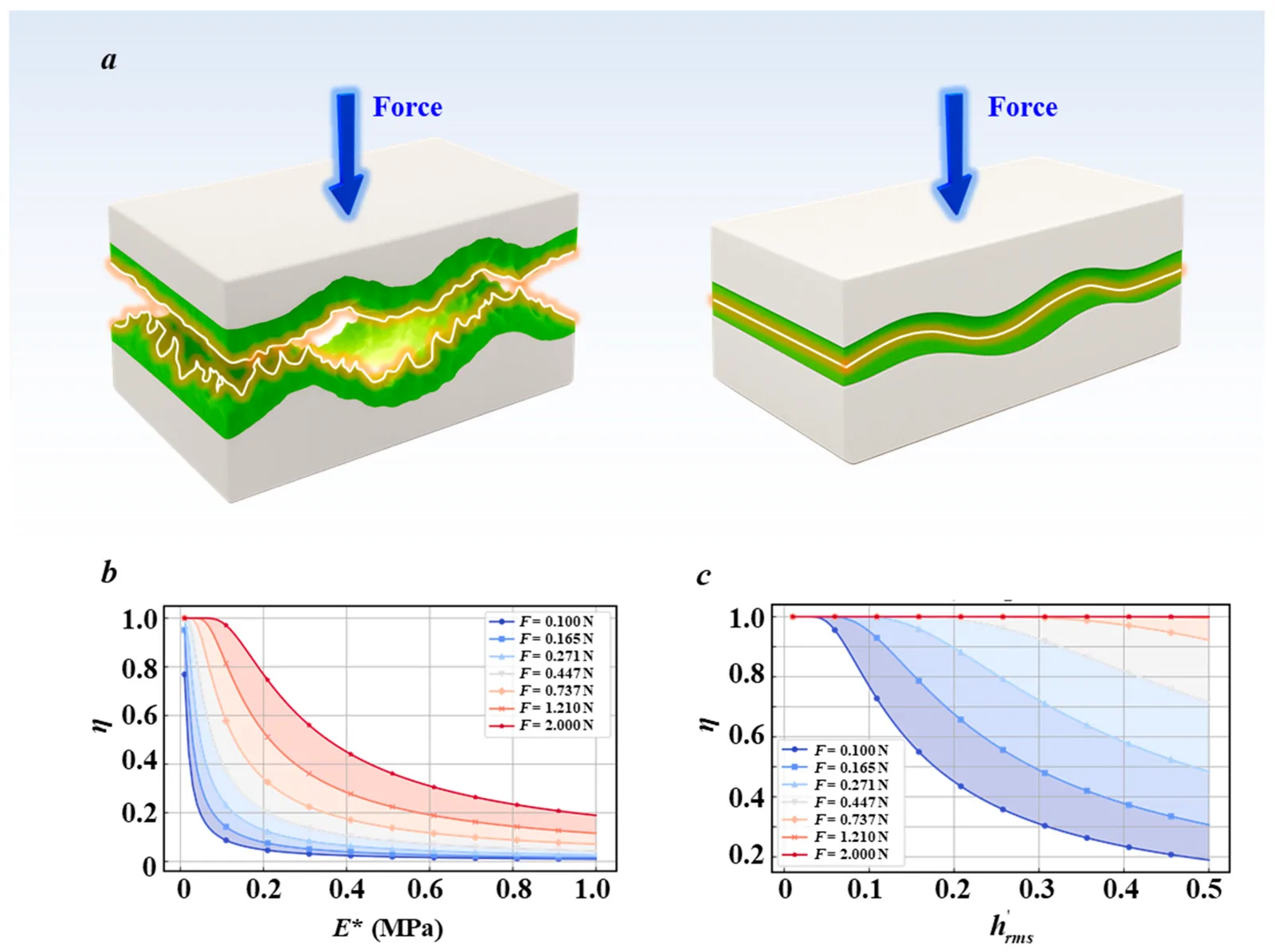
(**a**) Schematic of elastic contact between randomly rough surfaces. (**b**) Dependence of the real contact area on the effective elastic modulus and applied normal force. (**c**) Dependence of the real contact area on surface topography and applied normal force.

Figure 2a illustrates contact between two randomly rough elastic surfaces. As the normal load increases, the interfacial gaps decrease and the real contact area increases; the contact area therefore depends on surface roughness, mechanical properties, and applied load. Statistical rough-contact theories represent the surface as a random field and, within the elastic low-load regime, predict an approximately linear increase in real contact area with normal force [37,38,39]. Equation (2) adopts this mechanistic description to introduce a force-dependent real-contact fraction:

(2) $$\eta =A_{r}/A_{0}=\mathrm{erf}\left(\frac{\sqrt{2}}{2}\frac{\kappa F}{A_{0}h_{rms}^{’}E^{*}}\right)$$

Here, *η* is the real-contact fraction; *A*_r_ and *A*_0_ are the real and nominal contact areas, respectively; erf(·) is the error function; *κ* is a model-dependent constant of order unity; *F* is the applied normal force; E* is the effective contact modulus; and *h*^′^_rms_ is the root-mean-square slope of the combined surface topography. For a random surface *h*(*x*, *y*), the slope parameter is defined from the spatial gradient of the surface-height deviation from its mean value. Figure 2b shows that the calculated real-contact fraction increases with normal force and decreases with increasing effective modulus. Figure 2c shows that, at a given force, a rougher topography with a larger root-mean-square slope yields a smaller real-contact fraction.

For unchanged materials and environmental conditions, increasing the applied normal force enlarges the real contact area predicted by Equation (2). The larger contact area can increase the amount of triboelectric charge generated over the nominal interface; the effective surface-charge density is therefore modeled as a function of contact force, consistent with the cited experimental findings [40,41,42]. This relationship provides a mechanistic coupling between normal force and electrical response; it is not treated as an experimentally calibrated force-to-output transfer function in the present study. The effective surface-charge density is expressed as follows:

(3) $$\sigma =\eta \sigma_{s}$$

Here, *σ*_s_ is the surface-charge density corresponding to idealized complete contact and *σ* is the effective surface-charge density used in the equivalent electrical model.

**Figure 3:**
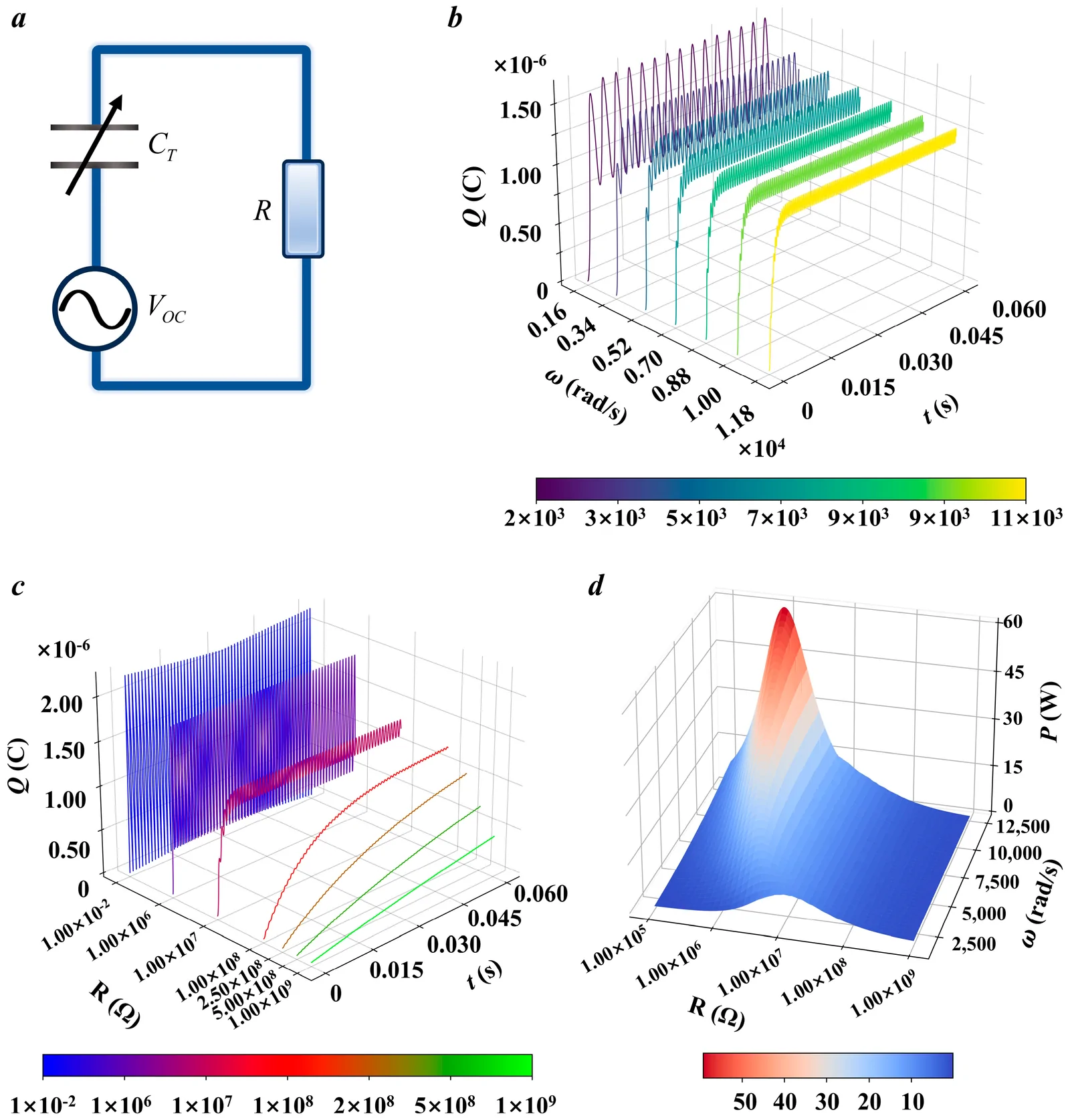
Theoretical parametric analysis based on the semi-analytical formulation. (**a**) Equivalent circuit of the RCSR-TENG connected to an external resistance. (**b**) Calculated transferred charge as a function of time at prescribed rotational speeds. (**c**) Calculated transferred charge as a function of time and external resistance. (**d**) Calculated output power as a function of external resistance and rotational speed. The calculations illustrate model trends and were not quantitatively fitted to the experiments.

The equivalent circuit of the RCSR-TENG (Figure 3a) comprises a time-dependent open-circuit voltage *V*_OC_, a total capacitance *C_T_*, and an external resistance *R*. At the initial fully overlapped state, the overlapped-region capacitance dominates because the dielectric thicknesses are much smaller than their in-plane dimensions. When the patterned regions approach complete separation, however, edge-field capacitance becomes increasingly important. The total effective capacitance is therefore written as follows:

(4) $$C_{T}\left(t\right)=\frac{\varepsilon_{0}A\left(t\right)}{d_{0}+h_{g}}+C_{ee}$$

where $d_{0}$ is the efficient thickness of the tribo-dielectric layers, $d_{0}=\frac{d_{1}}{\varepsilon_{r1}}+\frac{d_{2}}{\varepsilon_{r2}}$. $\varepsilon_{r1}$ and $\varepsilon_{r2}$ are the relative permittivity of dielectric 1 and dielectric 2, respectively. $d_{1}$ and $d_{2}$ are the thickness of dielectric 1 and dielectric 2, respectively. $h_{g}$ is the vertical gap between the tribo-surfaces. $C_{ee}$ is the effective capacitance now that the top plate and the bottom plate are fully separated.

Assuming ideal distribution of charge and a negligible electric field inside each electrode, the open-circuit voltage is obtained from Equation (5):

(5) $$V_{OC}\left(t\right)=\frac{\sigma \left[A_{\max}-A(t)\right]}{C_{T}(t)}$$

The governing equation for RCSR-TENGs also can be given as [28,43,44]

(6) $$R\frac{dQ}{dt}+\frac{Q}{C_{T}}-V_{OC}=0$$

where R and Q are the external resistance and the transferred charge between the bottom electrode and the top electrode. Substituting Equations (1)–(3) and (5) into Equation (6), an approximate semi-analytical V−Q−θ−F relationship was obtained. If RCSR-TENG work at a constant angle velocity rotation of ω, $\alpha \left(t\right)$ can be given by

(7) $$\alpha \left(t\right)=\omega t$$

The initial state is defined as the fully overlapped configuration after sufficient contact time for electrostatic equilibrium to be established between the two electrodes. The corresponding initial condition is

(8) $$Q\left(t=0\right)=0$$

Then, the output of the RCSR-TENG for arbitrary load resistance can be calculated as follows:

(9) $$Q(t)=\exp\left(-\int_{0}^{t}\frac{1}{C_{T}\left(\tau \right)R}d\tau \right)\times \int_{0}^{t}\frac{V_{OC}\left(\tau \right)}{R}\exp\left(\int_{0}^{\tau }\frac{1}{C_{T}\left(z\right)R}dz\right)d\tau$$

And then, the current, voltage and output power can be given as

(10) $$I(t)=\frac{dQ\left(t\right)}{dt}$$

(11) $$V(t)=I\left(t\right)R$$

(12) $$P(t)=V\left(t\right)I\left(t\right)$$

Replacing the previous piecewise angle functions with continuous functions removes artificial discontinuities at the segment boundaries and produces smoother parameter variation. This treatment can improve numerical convergence and reduce boundary-related numerical artifacts; it does not, however, establish predictive accuracy without independent identification and validation of the contact and electrical parameters.

Scope and parameter status of the formulation: Equations (1)–(12) constitute a forward semi-analytical calculation that combines rotation-dependent overlap, rough-contact mechanics, and the equivalent electrical circuit. The values listed in Table S1 are prescribed geometric, electrical, or swept parameters and were not obtained by fitting the experimental output. In particular, the force-dependent contact parameters were not independently measured for the fabricated device. Figure 3 therefore presents theoretical sensitivity and trend calculations rather than calibrated predictions of absolute device output.

All calculations use the fully overlapped state as the reference, for which the transferred charge is defined as *Q*(*t* = 0) = 0. To examine the effect of angular velocity on the calculated output, cases with different rotational speeds and external load resistances were evaluated using the prescribed parameters listed in Table S1. At a fixed load resistance, increasing the angular velocity shortens the period of each charge-transfer cycle. Although the calculated charge swing per cycle may decrease at higher speed, the larger time derivative d*Q*/d*t* produces a higher peak current; the corresponding resistive voltage and output therefore increase under the simulated conditions. Figure 3b–d presents the resulting transferred-charge and output-power trends.

## 3. Experiments and Simulations

### 3.1. Fabrication and Experimental Configuration

**Figure 4:**
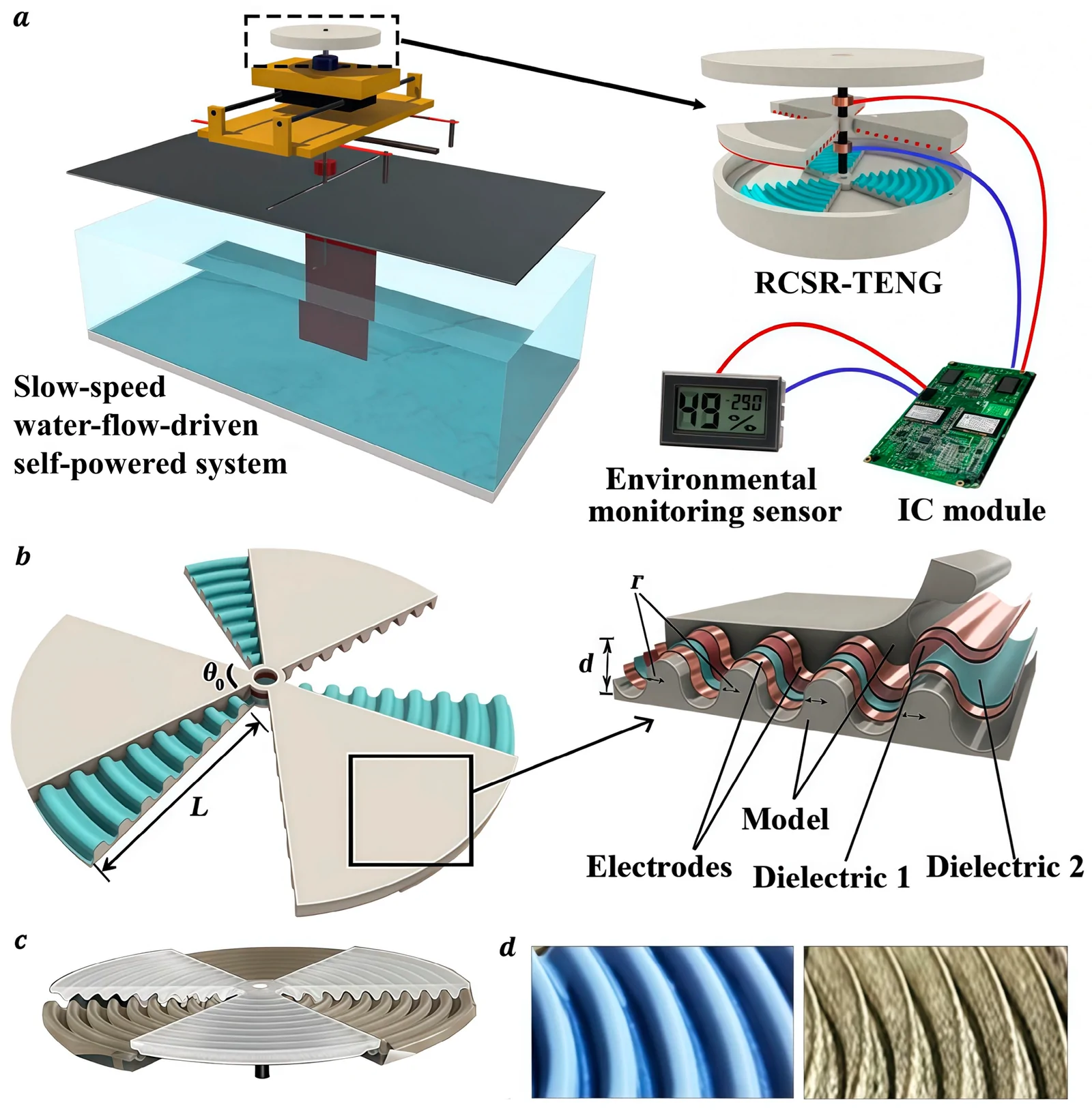
Structural design of RCSR-TENG and the slow-speed water-flow-driven self-powered system. (**a**) Schematic illustration of a slow-speed water-flow-driven self-powered system, which consists of a RCSR-TENG as the generator, a fully passive flapping foil driven by slow-speed water flow, an environmental-monitoring sensor as the load, and an IC module to store and manage the harvesting energy. (**b**) Expanded structural schematic of RCSR-TENG, which contains a rotator (conductive fabric as electrode 1, Teflon film as triboelectric material 1, a PMMA plate with riveted complementary structures) and a stator (a PMMA plate with riveted complementary structures, conductive fabric as electrode 2, and a ball bearing). (**c**) Optical photo of the RCSR-TENG. (**d**) Optical photos for Teflon film and conductive fabric fixed on the riveted complementary structure.

Figure 4a schematically depicts a proposed low-speed-water-flow-driven self-powered environmental-monitoring system comprising the RCSR-TENG, a passive flapping-foil collector, an integrated circuit (IC) for rectification and energy management, and a sensor with a Bluetooth module for wireless data transmission. In this conceptual architecture, the flapping motion is transmitted through the mechanical conversion mechanism and converted into rotor motion; the alternating electrical output would then be rectified, stored, and regulated before powering the sensor and communication module. The present experiments evaluated only the RCSR-TENG and its coupling to the passive flapping-foil collector. The IC, environmental sensor, and wireless communication blocks shown in Figure 4a were not incorporated into the tested prototype.

Layers of conductive fabric, polytetrafluoroethylene (PTFE) film, and conductive-fiber cloth were attached to the riveted complementary structures to form the electrodes and triboelectric layers, as shown in Figure 4b. These films were cut into fan-shaped pieces slightly larger than the corresponding structural units, allowing them to conform naturally to the curved profiles of the riveted complementary structures while providing sufficient margins for connection to electrical leads. The fabrication procedures for the RCSR-TENG and flat-plate R-TENG are described below. For the flat-plate R-TENGs, 5 mm thick acrylic sheets were cut into disks with an outer diameter of 150 mm, a central bore diameter of 6 mm for the drive shaft, and a second concentric bore diameter of 12 mm to accommodate bearings with an outer diameter of 12 mm and an inner diameter of 6 mm. The disks were machined using an automatic engraving machine ((Jinan Quick-Fulltek CNC Machinery Co., Ltd., Jinan, China). Sector-shaped regions with a central angle of 60° and annular-sector grooves with a central angle of 60° and a minimum radius of 8.5 mm were then machined alternately and at equal angular intervals to form the rotor and stator substrates.

For the RCSR-TENGs, the overall dimensions of the stators and rotors were identical to those of the corresponding flat-plate baseline design. The principal difference was that, after the sectors and grooves had been formed, an engraving bit with a diameter of 3 mm was used to machine the specified three-dimensional structures precisely along predefined paths. This process produced three distinct configurations: (1) a wavy geometry consisting of smoothly connected semicircular arcs with a radius of 1.5 mm; (2) a zigzag geometry consisting of continuous isosceles right triangles with a side length of 6 mm; and (3) a rectangular-wave geometry consisting of interconnected rectangular segments with a characteristic side length of 3 mm. To reduce mechanical abrasion during operation, the high-contact regions of the substrates were carefully polished, and their edges were smoothly chamfered.

Conductive fabric serving as the electrode material was attached to the surfaces of the acrylic stator substrates. Electrical leads were connected to the electrodes through extended tabs routed through predrilled holes in the backs of the substrates. For the rotor, electrical conduction was established by connecting the electrodes to the central shaft, which maintained electrical contact through the bearings. A 0.20 mm thick PTFE film and a 0.11 mm thick conductive-fiber-cloth film were firmly bonded to the electrode surfaces and served as the negative and positive triboelectric layers, respectively. These commercially available flexible materials were selected because they could conform readily to the three-dimensional acrylic profiles and were suitable for scalable fabrication.

Surface morphology was characterized using a ZEISS GeminiSEM 500 scanning electron microscope (Carl Zeiss Microscopy GmbH, Jena, Germany). Images of the conductive fabric and PTFE film are presented in Figure 4d, showing abundant microscale features on the conductive-fabric surface.

### 3.2. Numerical Simulation of Charge-Transfer Characteristics

A three-dimensional quasi-static electrostatic model was established in COMSOL Multiphysics 5.6 using the Electrostatics interface of the alternating current/direct current (AC/DC) Module. Each prescribed rotor angle was solved as an independent stationary state because electromagnetic wave-propagation effects are negligible at the operating frequencies considered.

**Figure 5:**
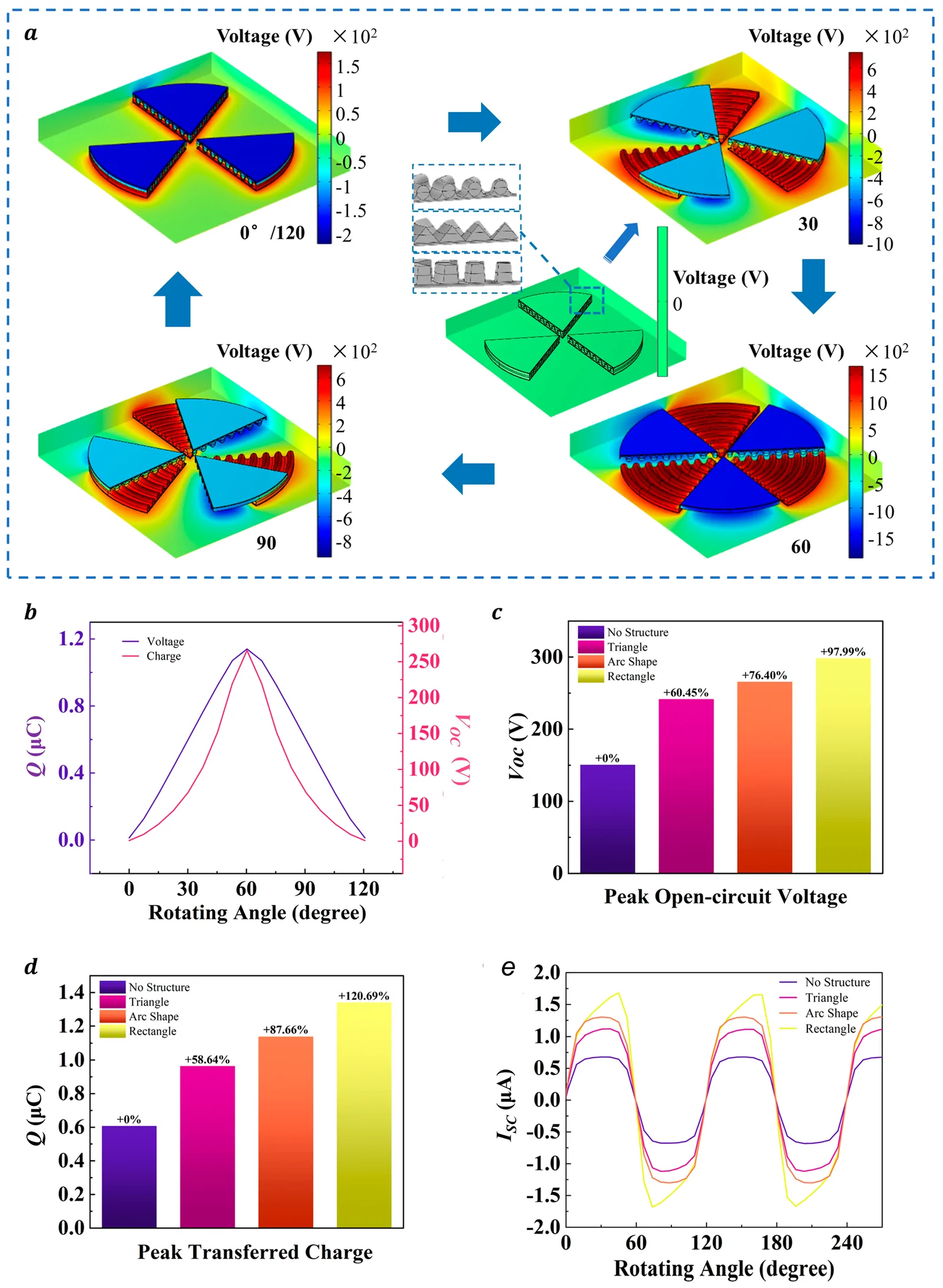
Numerical comparison of flat and riveted complementary structures. (**a**) Simulated electric-potential distributions of the arc-shaped RCSR-TENG at selected rotation angles. (**b**) Transferred charge and open-circuit voltage over one period. Comparison of (**c**) open-circuit voltage, (**d**) transferred charge, and (**e**) short-circuit current for the flat, triangular, arc-shaped, and rectangular profiles.

The idealized comparative model had an outer diameter of 100 mm. The curved-profile radius, center-to-center pitch, and axial-separation parameter were 1.5, 6, and 1 mm, respectively. The polyurethane (PU) and PTFE dielectric layers were each 1.7 mm thick, and the copper (Cu) electrodes were 0.3 mm thick. Relative permittivities of 5, 2, and 1 were assigned to PU, PTFE, and air, respectively, and the Cu conductivity was 5.81 × 10^7^ S m^−1^. The device was enclosed in a 300 × 300 × 100 mm^3^ air domain. In the archived comparison model associated with Figure 5, fixed surface-charge densities of −3.7795 × 10^−4^ and +3.7795 × 10^−4^ C m^−2^ were assigned to the opposed principal interfaces; the auxiliary independent-layer feature used +1.8898 × 10^−4^ C m^−2^. No free volume charge was assigned, and unassigned exterior boundaries used the default zero-charge condition.

Open-circuit voltage and short-circuit transferred charge were obtained in separate stationary studies. For the open-circuit calculation, the Cu electrodes were assigned independent Floating Potential conditions with zero initial net charge, and one exterior air boundary provided the electrical reference; *V*_OC_(*θ*) was the potential difference between the two floating electrodes. For the short-circuit calculation, both electrodes were grounded to realize the short-circuit condition, and COMSOL solved the electrostatic governing equations to obtain the induced charge on electrodes. By field-circuit coupling with the circuit module, COMSOL provided the transferred charge *Q*_tr_(*θ*), which characterizes the amount of charge flowing in the external circuit as the rotation angle *θ* varies. When the short-circuit current *I*_sc_ was plotted, it was calculated from the angle-dependent charge through *I*_sc_ = d*Q*_tr_/d*t* = *ω* d*Q*_tr_/d*θ* using the prescribed angular velocity *ω*.

The relative rotation angle was swept from 0 to 4π rad in increments of 0.04π rad (7.2°), giving 101 stationary states. Device domains used free tetrahedral elements, and the air domain used swept elements. The maximum and minimum element sizes were 24 and 3 mm, respectively; the maximum growth rate was 1.45, the curvature factor was 0.5, and the narrow-region resolution was 0.6. A representative open-circuit solution contained approximately 175,553 degrees of freedom. A fully coupled stationary solver was used with a relative tolerance of 1 × 10^−3^ and a maximum of 25 nonlinear iterations. The conjugate-gradient linear solver used a relative tolerance of 0.01, a maximum of 10,000 iterations, and a five-level algebraic-multigrid V-cycle preconditioner.

**Figure 6:**
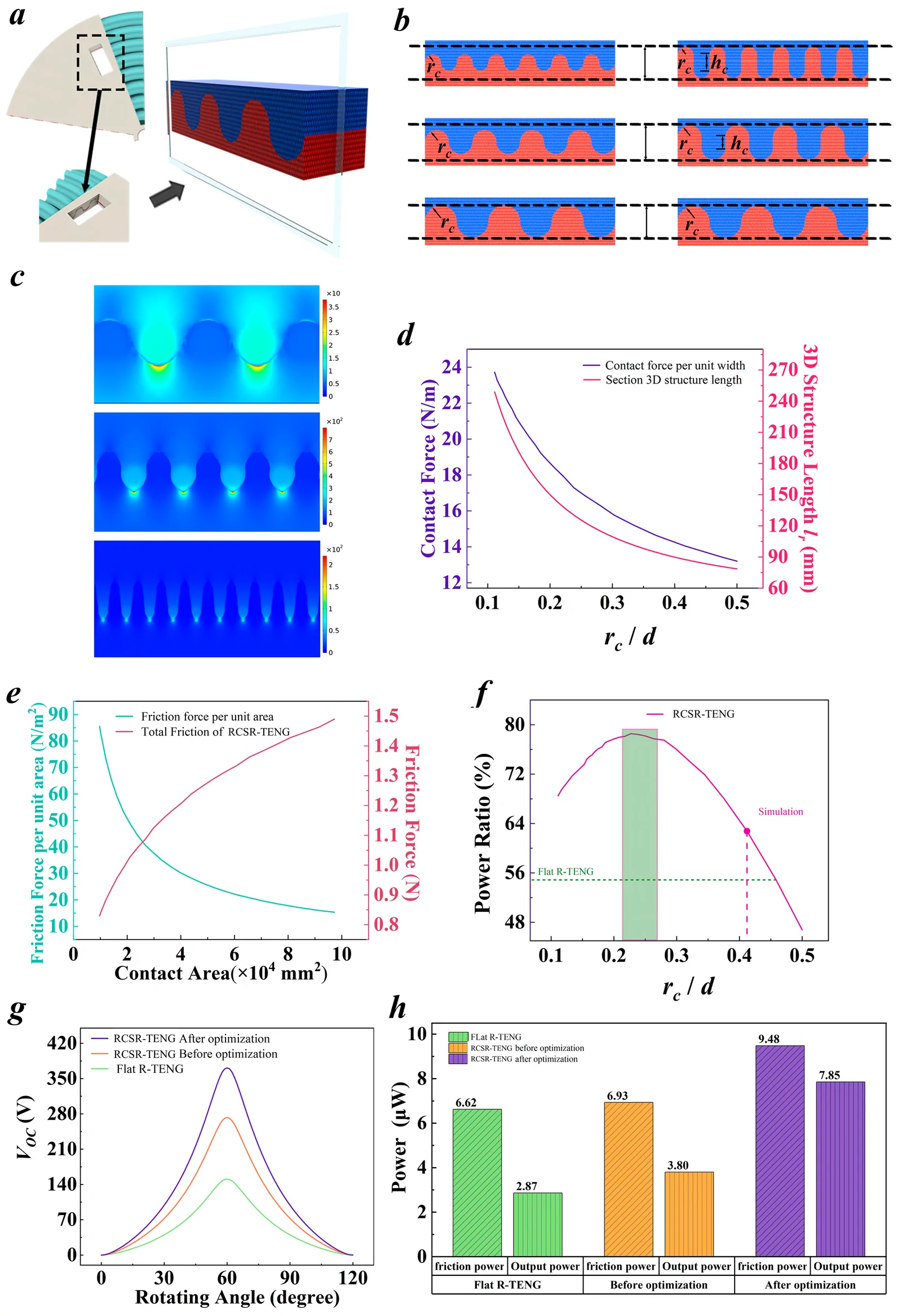
Structural optimization of the RCSR-TENG. (**a**) Location of the representative section and complementary profile before and after optimization. (**b**) Calculated real contact area *A*_r_ and specific-surface-area parameter *i* as functions of arc radius *r*_c_. (**c**) Stress distributions for representative *r*_c_/*d* ratios. (**d**) Equivalent profile length and normal contact force per unit width as functions of *r*_c_/*d*. (**e**) Total friction force and friction force per unit contact area as functions of real contact area. (**f**) Calculated electrical-output-to-friction-power ratio as a function of *r*_c_/*d*. (**g**) Open-circuit voltage before and after geometric optimization, compared with flat R-TENG. (**h**) Calculated frictional and electrical output powers for the flat, unoptimized, and optimized structures. The ratio in panel (**f**) is a model-based design metric.

**Figure 7:**
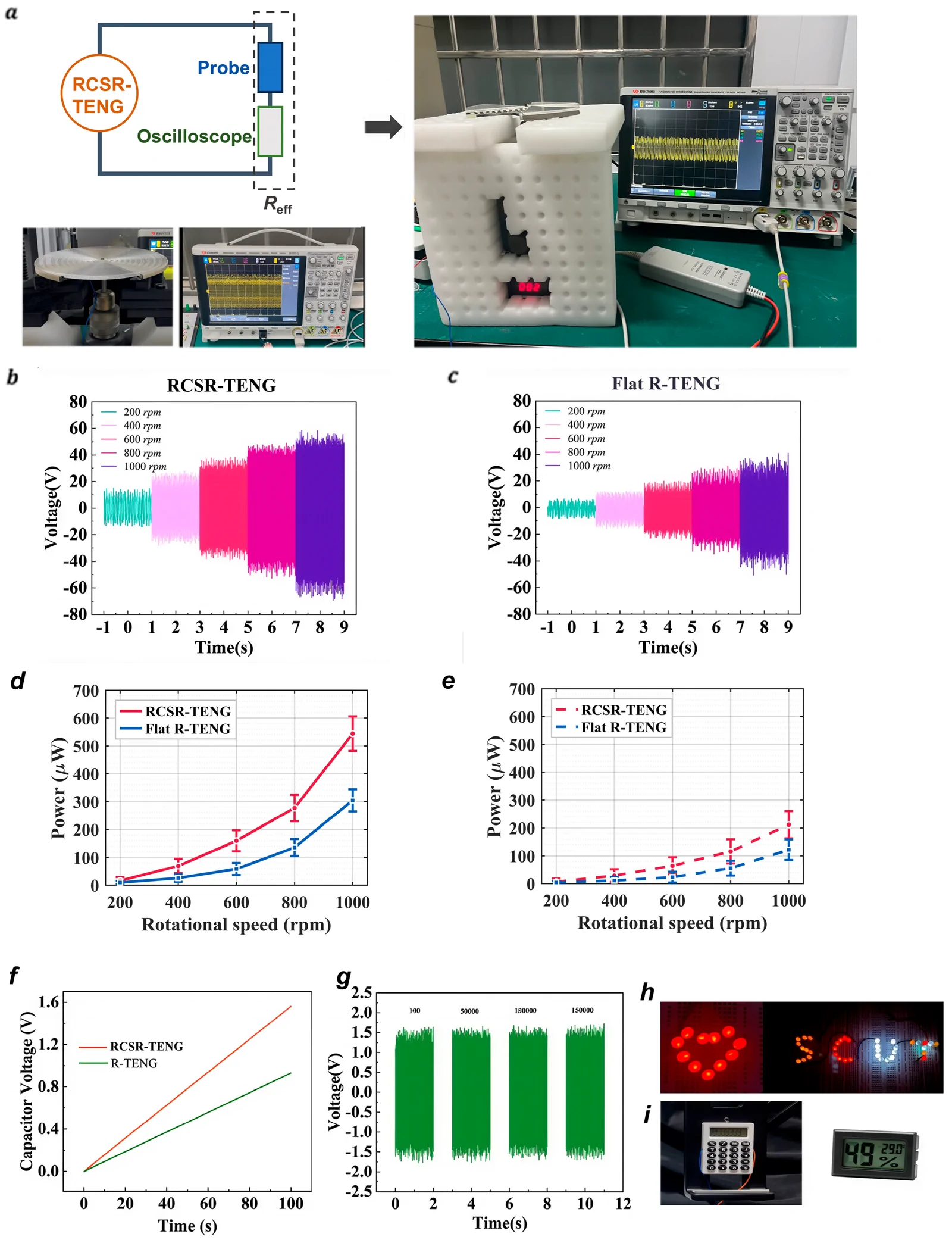
Electrical performance of the RCSR-TENG and flat R-TENG prototypes. (**a**) Measurement circuit and apparatus. Voltage waveforms of (**b**) the RCSR-TENG and (**c**) the flat R-TENG at 200–1000 rpm. (**d**) Peak output power at different rotational speeds. (**e**) Time-averaged output power at different rotational speeds. (**f**) Charging a 100 μF capacitor at 600 rpm. (**g**) Voltage waveforms recorded over 150,000 rotation cycles. (**h**) Illumination of 37 series-connected light-emitting diodes (LEDs). (**i**) Demonstrations powering a calculator and a temperature/humidity display. One prototype of each configuration was tested (*n* = 1 per configuration); the curves do not represent independently fabricated-device statistics.

**Figure 8:**
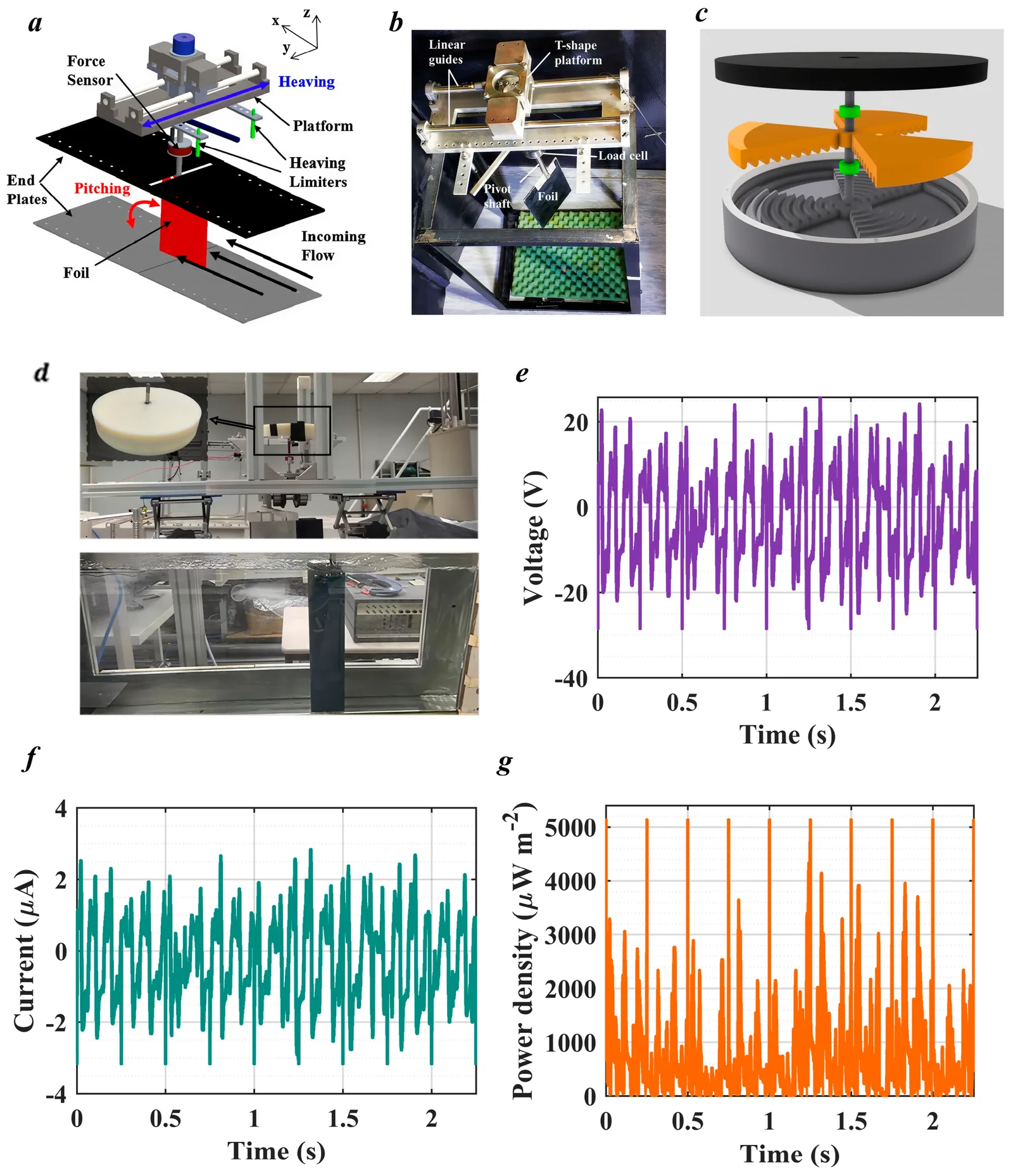
Low-speed water-flow energy harvesting using the RCSR-TENG and a passive flapping-foil collector. (**a**) Structural schematic of the collector, in which the adjustable heaving and pitching limiters passively constrain the motion range. (**b**) Photograph and components of the collector; the force sensor and load cell were used for experimental characterization only and were not components of a closed-loop damage-protection system. (**c**) Three-dimensional schematic of the packaged RCSR-TENG and transmission. (**d**) Integrated test setup. Measured (**e**) voltage, (**f**) current, and (**g**) area-normalized power of the RCSR-TENG driven by the passive flapping-foil collector.

The COMSOL model was constructed as an idealized comparative model to isolate the influence of surface-profile geometry under identical electrical and material parameters; it was not calibrated as a dimensional or material replicas of the fabricated prototype. In the model, 1.7 mm polyurethane (PU) and 1.7 mm polytetrafluoroethylene (PTFE) layers were used as the positive and negative triboelectric domains, respectively, and 0.3 mm copper (Cu) layers were treated as homogeneous, equipotential electrodes. The same material stack and thicknesses were applied to the flat, triangular, arc-shaped, and rectangular models so that the calculated differences could be attributed to the surface-profile geometry. By contrast, the experimental prototype employed a 0.20 mm PTFE negative triboelectric layer, a 0.11 mm conductive-fiber-cloth positive triboelectric layer, and flexible conductive-fabric electrodes. Consequently, the absolute simulated electrical quantities are used only for comparative geometry screening and are not interpreted as quantitative predictions of the fabricated device. Figure 5 and Figure 6 present deterministic numerical or analytical calculations and therefore do not include experimental error bars. The prototype comparison in Figure 7 used one RCSR-TENG and one flat R-TENG (*n* = 1 per configuration). The water-flow demonstration in Figure 8 used one integrated RCSR-TENG/flapping-foil assembly. Consequently, the experimental curves should be interpreted as proof-of-concept, within-device results rather than as statistics across independently fabricated devices.

## 4. Results and Discussion

### 4.1. Influence of Riveted Complementary Structures on Electrical Output

Figure 5a shows the simulated potential distributions of the arc-shaped RCSR-TENG at rotation angles of 0°, 30°, 60°, 90°, and 120° (equivalent to the next 0° position), respectively. At 0°, the two triboelectric regions are fully overlapped, and the potential difference between the upper and lower electrodes is approximately zero. As the rotor moves from 0° to 60°, the potential difference increases; as it moves from 60° to 120°, the potential difference decreases toward zero. Figure 5b shows the corresponding variations in transferred charge and open-circuit voltage over one rotation period. Both quantities increase from zero to a peak and then return to zero. The peak transferred charge and open-circuit voltage are 1.13 μC and 266 V, respectively. The short-circuit transferred charge was obtained by electrically connecting the two electrodes through a negligible resistance in the model and likewise varies periodically with rotation angle.

Triangular and rectangular complementary surface profiles were also modeled. Figure 5c–e compares the simulated electrical outputs of the four geometries. The flat R-TENG, denoted as “No Structure” and used as the reference, produced peak transferred charge and open-circuit voltage values of approximately 0.60 μC and 151 V, respectively. Relative to the flat reference, the triangular, arc-shaped, and rectangular structures increased the open-circuit-voltage peaks by 60% (242 V), 76% (266 V), and 98% (298 V), respectively (Figure 5c). The corresponding transferred-charge increases were 59% (0.96 μC), 87% (1.13 μC), and 121% (1.34 μC), respectively (Figure 5d). As shown in Figure 5e, the flat reference produced a peak short-circuit current of 0.68 μA, whereas the triangular, arc-shaped, and rectangular structures increased the peak values by 63% (1.11 μA), 95% (1.33 μA), and 148% (1.68 μA), respectively.

These idealized numerical results indicate that the three-dimensional complementary profiles can improve the calculated electrical output relative to a flat geometry under identical model settings. The arc-shaped and rectangular profiles performed particularly well. The arc-shaped structure also exhibited no pronounced localized stress concentration under the simulated conditions, suggesting a potential wear-resistance advantage. Accordingly, the subsequent optimization combines the smooth curvature of the arc-shaped profile with the increased contact area associated with a more compact complementary geometry.

### 4.2. Structural Optimization of the RCSR-TENG

The actual contact area of the triboelectric layers strongly influences the electrical output of the RCSR-TENG. Because of the device geometry, the longitudinal section of the arc-shaped complementary profile was selected to establish the structural-optimization model, as shown in Figure 6a. In this model, the complementary rotor and stator surfaces were assumed to be in conformal contact.

As shown in Figure 6b, the calculated contact area *A*_r_ and specific-surface-area parameter *i* both increase as the arc radius *r*_c_ decreases within the investigated geometric range. The increase in available contact area can promote charge generation and improve the calculated electrical output. Stress distributions were then calculated for three representative values of the dimensionless geometric ratio *r*_c_/*d*, as shown in Figure 6c. Figure 6d shows that decreasing *r*_c_/*d* increases both the equivalent profile length and the normal contact force per unit width. The corresponding friction force was estimated from the calculated normal contact force using the coefficient of friction specified in the model. Figure 6e shows that, as the actual contact area increases, the calculated total friction force increases, whereas the friction force normalized by contact area decreases.

To balance the increased contact area against the additional frictional demand, Figure 6f plots the calculated electrical-output-to-friction-power ratio as a function of *r*_c_/*d*. At large values of *r*_c_/*d*, the relatively small contact area and low specific-surface-area parameter limit the calculated electrical output. By contrast, excessively small values produce closely spaced complementary features and substantially increase the frictional demand. A ratio of *r*_c_/*d* = 0.25 was therefore selected as a compromise. At this value, the calculated open-circuit voltage was 34.0% higher than that of the unoptimized complementary structure and 146% higher than that of the flat structure (Figure 6g). Figure 6h shows that optimization increased the calculated frictional power from 6.93 to 9.48 μW (36.8%) and the calculated electrical output power from 3.80 to 7.85 μW (106.6%). Relative to the flat structure, which produced a calculated electrical output power of 2.87 μW, the optimized complementary structure produced 173.5% higher output power. The calculated electrical-output-to-friction-power ratios were 43.3%, 54.8%, and 82.8% for the flat, unoptimized complementary, and optimized complementary structures, respectively. This ratio is a model-based design metric and should not be interpreted as the complete mechanical-to-electrical conversion efficiency because shaft input power and parasitic mechanical losses were not included in the adopted model boundary.

### 4.3. Electrical Performance of the RCSR-TENG

To clarify the relationships between rotational speed, electrical output, and the enhancement provided by the three-dimensional complementary structure, the alternating load voltage and current of the RCSR-TENG with an arc-shaped complementary structure and those of the flat R-TENG were measured using the circuit shown in Figure 7a. An IKA EUROSTAR 20 digital overhead stirrer (IKA-Werke GmbH & Co. KG, Staufen, Germany) was used as the rotary drive. The output voltage was recorded using a Keysight InfiniiVision 4000 X-Series oscilloscope equipped with an N2790A high-voltage differential probe (Keysight Technologies, Inc., Santa Rosa, CA, USA). The voltage signal in the rotational tests was recorded using an oscilloscope equipped with a differential probe. Under the experimental wiring configuration, the 8 MΩ input resistance of the differential probe and the 1 MΩ oscilloscope input resistance were represented as a series-equivalent external measurement load of *R*_eff_ = 9 MΩ.

(13) $$P_{\mathrm{peak}}=\frac{V_{\mathrm{peak}}^{2}}{R_{\mathrm{eff}}}$$

The time-averaged power was calculated as

(14) $$P_{\mathrm{avg}}=\frac{1}{T}\int_{0}^{T}\frac{v^{2}(t)}{R_{\mathrm{eff}}}dt$$

Temperature and relative humidity were monitored using a commercially available TH20R temperature and humidity meter (Shenzhen Huahanwei Technology Co., Ltd., Shenzhen, China). During the electrical measurements, the room temperature was maintained above 25 °C and the ambient relative humidity remained below 60%. The rotational speed varied from 200 to 1000 rpm at intervals of 200 rpm. The corresponding rotational angular speed ranged from ~21 rad/s to 105 rad/s. As shown in Figure 7b,c, the measured load-voltage amplitude of the RCSR-TENG increased from approximately 10 V at 200 rpm to approximately 70 V at 1000 rpm. The flat R-TENG produced a lower load voltage at each tested speed. The current values used for the rotational power calculation were derived from the measured voltage and the fixed effective resistance of 9 MΩ; they were not measured independently.

The corresponding peak output power is presented in Figure 7d. The output power of both RCSR-TENG and R-TENG increases with rising rotation speed. As reported in the recent rotating-type TENG work [45], the output power of such devices varies with external load resistance. The external resistance is 9 MΩ, the peak output power of RCSR-TENG can reach 544 μW at 1000 rpm (Figure 7d), which corresponds to a ~79% enhancement compared with R-TENG.

Figure 7e compares the average output powers of the two prototypes calculated under the reported effective 9 MΩ measurement load. At 1000 rpm, the average output powers reached 211 μW for the RCSR-TENG and 122 μW for the flat R-TENG. Thus, under the same reported measurement condition, the RCSR-TENG produced 73% higher calculated power than the flat R-TENG. This comparison was obtained under a fixed effective measurement load and should not be described as a matched-load result because an experimental resistance sweep was not performed.

After rectification, the RCSR-TENG charged a 100 μF capacitor from 0 to approximately 1.6 V within 100 s at 600 rpm. Under the same charging conditions, the final capacitor voltage obtained using the RCSR-TENG was approximately 0.67 V higher than that obtained using the flat R-TENG, as shown in Figure 7f. As shown in Figure 7g, the recorded voltage waveform showed no obvious decrease in amplitude over 150,000 rotation cycles. This result demonstrates the within-device operational stability of the tested prototype but does not independently establish material degradation behavior or device-to-device reproducibility. After rectification and connection to an energy-storage circuit, the RCSR-TENG illuminated 37 series-connected light-emitting diodes and powered a calculator and a temperature/humidity display, as shown in Figure 7h,i. These demonstrations establish the proof-of-concept capability of the device to charge a capacitor and power low-power electronic devices under the tested conditions. One RCSR-TENG prototype and one flat R-TENG prototype were tested; therefore, the reported results do not represent independently fabricated-device statistics.

## 5. Application for Low Speed Waterflow Energy Harvesting

A passive flapping-foil collector was coupled to the RCSR-TENG to examine low-speed water-flow energy harvesting, as shown in Figure 8a–c. The collector comprised a rigid foil measuring 140 mm in chord, 200 mm in span, and 5 mm in thickness, with a mass of 0.25 kg. The foil was mounted vertically on a 0.9 kg translating platform through a shaft and bearings. Under hydrodynamic loading, the foil underwent self-excited coupled heaving and pitching without an active actuator. The upper T-shaped carriage was constrained by two linear guides to translate along the prescribed heave direction, while the foil shaft transmitted the pitching motion. This passive mechanism simplifies the external drive requirement [46,47,48,49], although the complete system still contains bearings, guides, limiters, and a mechanical transmission. The packaged assembly converts the foil’s oscillatory motion into periodic electrical output from the RCSR-TENG.

Large-amplitude hydrofoil motion was constrained by two pairs of passives, position-adjustable mechanical limiters, as shown in Figure 8a,b. The lower pair was installed beneath the linear guides and periodically contacted the long arm connected to the vertical hydrofoil shaft when the translating platform approached either end of its heaving stroke. The resulting mechanical constraint promoted hydrofoil pitching and stroke reversal, thereby bounding the heaving displacement. The upper pair was positioned near the top of the shaft and contacted the short arm when the hydrofoil approached the prescribed pitch boundary, preventing further angular rotation. The positions of both limiter pairs were adjusted before each experiment to define the allowable heaving and pitching ranges. Thus, the limiters controlled the maximum motion through direct mechanical contact and were neither sensor-actuated nor operated in a closed-loop manner. The force sensor and load cell shown in Figure 8b were used only for experimental characterization; they were not components of an active damage-protection system.

Figure 8d and Video S1 show the assembled RCSR-TENG/flapping-foil prototype in the recirculating water channel. The channel test section measured 2.0 m in length, 0.3 m in width, and 0.6 m in height and provided nominally uniform flow at velocities up to 1.0 m s^−1^. For the proof-of-concept test, the flow velocity was set to 0.55 m s^−1^. At a water-flow velocity of 0.55 ms^−1^, the foil oscillated at approximately 0.5 Hz, with a peak-to-peak pivot-axis displacement of 160 mm, a pitching amplitude of 45°, and a peak torque of 1.2 N·m. For a foil span of *b* = 200 mm, the swept area was 2*h*_0_·*b* = 0.032 m^2^, and the hydraulic input power was approximately 2.3 W. The losses comprised hydraulic, volumetric, and mechanical components; the mechanical transmission losses were approximately 6%, compared with hydraulic losses exceeding 60%. The submerged foil drove the vertical shaft through self-excited heaving and pitching, and the mechanical transmission converted the oscillatory motion into rotation of the RCSR-TENG. The peak area-normalized power was exceeding 5000 μW m^−2^. The time-averaged power was calculated from the instantaneous voltage and current measured synchronously under the same load condition:

(15) $$d_{\mathrm{avg}}=\frac{\frac{1}{T}\int_{0}^{T}\left|v(t)i(t)\right|dt}{A}1$$

(16) $$A=\pi {\left(\frac{0.150\;m}{2}\right)}^{2}=\pi {\left(0.075\;m\right)}^{2}=1.767\times 10^{-2}\;m^{2}$$

where *A* is the projected device area. The average value is 875 μW m^−2^. The reported area-normalized power uses the projected RCSR-TENG footprint as the normalization area; it is not a water-flow-to-electrical conversion-efficiency measurement.

Compared with a conventional rotary turbine, the fully passive flapping-foil collector avoids centrifugal loading and operates with a relatively low tip speed, while its approximately rectangular swept area is advantageous in wide, shallow channels [46,47,48]. These attributes make the collector a suitable mechanical interface for low-velocity flows. The present results demonstrate that the RCSR-TENG can be driven by such a collector and can produce periodic electrical output. However, complete water-flow-to-electrical efficiency was not measured, and the demonstration should therefore be interpreted as proof of concept rather than as evidence of superior system-level conversion efficiency.

## 6. Conclusions and Outlook

This study developed a rotating triboelectric nanogenerator with three-dimensional riveted complementary surface structures for low-speed water-flow energy harvesting. The complementary profiles increased the available contact area and provided circumferential guidance for the rotor. A semi-analytical formulation incorporating a force-dependent real-contact fraction was used to examine the coupled effects of rotation, contact, and the external electrical circuit. Because its contact parameters were not independently calibrated, the formulation provides sensitivity and trend information rather than quantitatively validated predictions. Comparative simulations showed that the three-dimensional profiles increased the calculated open-circuit voltage, transferred charge, and short-circuit current relative to flat geometry. For the single prototype tested for each configuration, the RCSR-TENG produced peak and time-averaged output powers of 544 and 211 μW, respectively, at 1000 rpm under the fixed effective measurement load of 9 MΩ. Under the same conditions, the corresponding values for the flat R-TENG were 304 and 122 μW, representing increases of approximately 79% and 73%, respectively.

The tested RCSR-TENG also maintained a stable voltage amplitude over 150,000 rotation cycles. Coupling the generator to a fully passive flapping-foil collector produced periodic voltage, current, and area-normalized power under a water-flow velocity of 0.55 m s^−1^, demonstrating proof-of-concept applicability to low-speed water-flow energy harvesting. The present results establish the structural and electrical advantages of the RCSR design under the tested conditions; they do not yet establish complete mechanical-to-electrical efficiency, water-flow-to-electrical efficiency, or autonomous environmental-sensor operation.

Future work should identify the contact and electrical parameters through independent measurements and validate the formulation over controlled normal force, external resistance, and rotational speed using a force–resistance–speed test matrix with predefined prediction-error metrics. A prototype-calibrated multiphysics model should incorporate the measured dielectric, conductive, surface-charge, deformation, and contact properties of the flexible material stack. Multiple independently fabricated devices should be tested for each geometry, with device-level means, dispersion, confidence intervals, and predefined statistical comparisons. Complete shaft-to-electrical and water-flow-to-electrical efficiencies should be determined from synchronized input- and output-power measurements with clearly defined system boundaries. To progress toward autonomous environmental monitoring, the RCSR-TENG should be integrated with low-leakage rectification, impedance matching, intermediate energy storage, voltage regulation, sensor duty-cycle control, signal processing, and wireless communication. Long-term water operation will additionally require waterproof interconnections, corrosion-resistant electrodes, antifouling protection, and mechanically isolated packaging, while the mass, stiffness, and hydrodynamic resistance introduced by the integrated components must be included in the energy system budget. Finally, non-contact displacement and angular-position sensors; impact-force or acceleration monitoring; and a threshold-based controller capable of issuing an alarm, increasing damping, or shutting down the system should be added because the present passive limiters are not a fail-safe damage-protection system.

## Data Availability

The original contributions presented in this study are included in the article/Supplementary Material. Further inquiries can be directed to the corresponding authors.

## Supplementary Materials

The following supporting information can be downloaded at: https://www.mdpi.com/article/10.3390/ma19173569/s1.
