# Supplementary material for: High-Performance Riveted Complementary-Structure Rotating Triboelectric Nanogenerator for Energy Harvesting from Slow-Speed Water Flows
Source: Materials (Basel). 2026 Aug 22;19(17):3569. doi: 10.3390/ma19173569 (PMC13566815; doi:10.3390/ma19173569)
Supplement: Supplementary file 1 [file materials-19-03569-s001.zip › materials-4474544-supplementary.pdf]

Supporting Information

High Performance Riveted Complementary Structure Rotating Triboelectric Nanogenerator for Energy Harvesting from Slow-speed Water Flows

Bao Yang [1], Chang Peng [1], Zihao Wang [1], Fuwang Zhao [2], Licheng Zhou [1], Zhenyu Jiang [1], Yiping Liu [1], Liqun Tang [1], Zejia Liu [1,*], Jinli Piao [3,*]

*Corresponding e-mails: zjliu@scut.edu.cn; jinlifw.piao@connect.polyu.hk

Section S1. Derivation of the Nominal Contact Area of the Rotating Triboelectric Nanogenerator with Riveted Complementary Structures (RCSR-TENG)

As shown in Figure S1a, the upper component rotates clockwise with the central axis of the RCSR-TENG. The rotation angle is θ, and r_1 and r_2 are the inner and outer radii of a fan-shaped unit, respectively. The complementary contact profile is formed from semicircular features of radius r_c and line segments of length h_l. The corresponding numbers of semicircular features and line segments are n_c and n_l, respectively. The maximum nominal contact area is given by:

$$A_m = N\int_0^{\theta_c} \frac{1}{2}\left(r_1 + n_c\pi r_c + n_l h_l\right) r_2 \; d\theta - N\int_0^{\theta_c} \frac{1}{2} r_1^2 \; d\theta \tag{S1}$$

For the semicircular-arc geometry used in the design, the following relation applies:

$$2n_c r_c = r_2 - r_1 \tag{S2}$$

Combining Equations (S1) and (S2) gives:

$$A_{\mathrm{m}} = \frac{\pi}{2}\left[\left(r_1 + \frac{\pi}{2} r_2 - \frac{\pi}{2} r_1 + n_l h_l\right) r_2 - r_1^2\right] \tag{S3}$$

Because the nominal contact area varies with rotation, it is expressed as:

$$A(t) = A_{\mathrm{m}}\left[1 - \frac{1}{\pi}\arccos\left(\cos\left(N\alpha(t)\right)\right)\right] \tag{S4}$$

For a flat-plate rotating triboelectric nanogenerator, the corresponding expression is obtained by setting the profile radius r_c and the line-segment length h_l to zero.

Section S2. Comparison of the Contact Area between the RCSR-TENG and Flat-Plate Rotating Triboelectric Nanogenerator (R-TENG)

Figure S1(a) shows representative calculated electric-potential distributions of the flat-plate R-TENG at successive rotation angles. The potential difference varies periodically as the overlap between the triboelectric layer's changes. Figure S1b compares the nominal contact areas of the RCSR-TENG and flat-plate R-TENG; the label "F-TENG" in panel (b) denotes the flat-plate R-TENG reference. Within the geometric model, the complementary features increase the nominal contact area relative to the flat-plate geometry, and the magnitude of this increase depends on the semicircular-feature radius and line-segment length. These results are deterministic theoretical comparisons based on the prescribed parameters in Table S1 and should not be interpreted as experimental measurements or quantitatively calibrated predictions of the fabricated device.

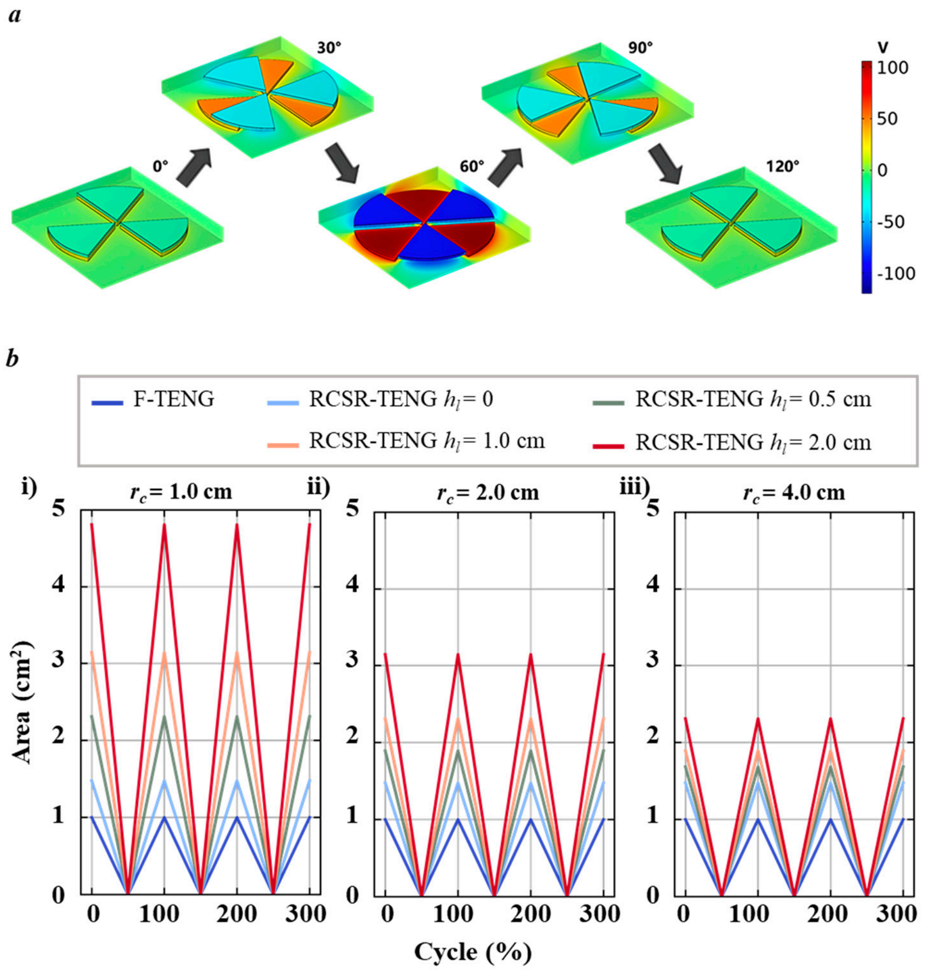

Figure S1. (a) Representative calculated electric-potential distributions of the flat-plate R-TENG at successive rotation angles. (b) Theoretical comparison of the nominal contact areas of the RCSR-TENG and flat-plate R-TENG for the parameter sets indicated in the figure; “F-TENG” denotes the flat-plate R-TENG reference. The calculations illustrate geometric trends and were not quantitatively fitted to the experiments.

Section S3. Parameters Used in the Theoretical Model

Table S1. Parameters used in the theoretical model of the RCSR-TENG.

Parameter	Value
External resistance, R	9 MΩ
Surface triboelectric charge density, σ	50 μC m^{-2}
Thickness of the dielectric layer, d_1	200 μm
Relative permittivity of the dielectric layer, ε_{r_1}	3.4
Effective thickness of the dielectric layer, d_0	58.82 μm
Number of fan-shaped units, N	3
Central angle, θ_c	π/3 rad (60°)
Inner radius of the fan-shaped unit, r_1	2 cm
Outer radius of the fan-shaped unit, r_2	7.5 cm
Rotational speed	200–2000 rpm
Length of the line segments, h_l	0–2 cm
Number of line segments, n_l	0–8
Radius of the semicircular features, r_c	0–4 cm
Number of semicircular features, n_c	0 or $(r_2 - r_1)/(2r_c)$
Vacuum permittivity, ε_0	8.854×10^{-12} F m^{-1}
